# Frequency of Caesarean Section Classified by Robson’s Ten Group Classification System: A Scoping Review

**DOI:** 10.7759/cureus.41091

**Published:** 2023-06-28

**Authors:** Farah Jiandani, Savita Somalwar, Anuja Bhalerao

**Affiliations:** 1 Department of Obstetrics and Gynaecology, NKP Salve Institute of Medical Sciences and Research Centre, Nagpur, IND

**Keywords:** caesarean delivery, classification system, antenatal women, scoping review, robson’s ten group

## Abstract

The prevalence of caesarean section (CS) is rising rapidly. However, it should be carried out only under valid obstetric indications due to the various complications associated with it. Therefore, to record CS incidences, Robson’s Ten Group Classification System (TGCS) was implemented. This review focuses on the prevalence of CS rates found in various studies and identifies the clinically important groups that were most involved in CS deliveries. Preferred Reporting Items for Systematic Review and Meta-Analysis Extension for Scoping Review (PRISMA-ScR) guidelines were followed in this review. "Caesarean delivery" OR "Robson's Ten Group" OR "Classification System" were keywords used to search literature. Twenty-one studies were included in this review based on eligibility criteria. We concluded that group 5 was the major contributing factor for the increase in CS rates followed by group 10, group 4, group 2, and group 1. Previous CS was the most common factor responsible for increasing CS rates. We emphasize that Robson's TGCS is an essential parameter for recording CS rates and is simple to use for CS rate comparison.

## Introduction and background

Caesarean section (CS) rates that are population-based should ideally be 10-15% as per the World Health Organization (WHO) [1]. Even so, both developed and developing nations are seeing an increase in the frequency of CS. The latest data show that India's CS rate is 17.2% at the population level, whereas it represents 21% of births globally [2]. CS should only be performed when there is a valid obstetrical indication under suitable conditions that indicates that it will reduce maternal or neonatal morbidity and mortality [3]. CS is lifesaving when vaginal deliveries pose a risk but not all CS deliveries are necessary and needed. CS is associated with various complications such as prolonged hospital stay, post-partum haemorrhage, blood transfusion, retained placenta, and post-partum infections. CS is more common in urban areas compared to rural due to easy access to healthcare in urban sectors [4,5].

Recording CS rates, incorporating efficient strategies for optimizing CS rates, advancements in treatment procedures, as well as better patient supervision are all important initiatives that lower CS prevalence and ensure the safety of both mother and child. WHO in 2015 and the International Federation of Gynecology and Obstetrics (FIGO) in 2016 implemented Robson's Ten Group Classification System (TGCS) as a universal benchmark to evaluate, compare, and monitor CS occurrence within healthcare institutions, between facilities, and over time.

According to this theoretical framework, all women fall under one of 10 groups that are comprehensive and mutually exclusive. The categorization is founded on five basic obstetrical features: prior CS, parity, number of fetuses, gestational age, the start of labour, and fetal presentation [2,6,7]. The aim of this review was to assess and analyse the incidence of CS identified in different studies as well as to determine which clinically significant populations primarily accounted for the administration of CS since very few investigations examined CS rates using Robson's categorization.

## Review

This review followed Preferred Reporting Items for Systematic Review and Meta-Analysis extension for Scoping Review (PRISMA-ScR) guidelines. The inclusion criteria consisted of articles with full-text availability, giving information regarding the frequency of caesarean delivery classified based on Robson’s TGCS, published from 2018 onwards, and in the English language. Articles not providing brief descriptions regarding the frequency of caesarean delivery classified based on Robson’s TGCS, articles published before 2018, and articles that were not published in the English language were excluded from the study. An aggregate of 21 research papers was included in this review after being assessed for inclusion. Robson's TGCS is described in Table 1 [2].

**Table 1:** Robson's ten group classification system CS: Caesarean section Source: Pravina et al., 2022 [2].

Group	Classification
Group 1	Nulliparous, single, cephalic pregnancy > 37 weeks in spontaneous labour
Group 2	Nulliparous, single, cephalic pregnancy > 37 weeks who had labour induced or delivered before labour by CS
Group 3	Multiparous, without previous uterine scar with single, cephalic pregnancy > 37 weeks in spontaneous labour
Group 4	Multiparous, without previous uterine scar with single, cephalic pregnancy > 37 weeks who had labour induced or delivered before labour by CS
Group 5	All multiparous with at least one previous uterine scar, with single cephalic pregnancy > 37 weeks
Group 6	All nulliparous with a single breech pregnancy
Group 7	All multiparous with a single breech including women with previous scars
Group 8	All women with multiple pregnancies including those with uterine scars
Group 9	All women with a single pregnancy with transverse or oblique lie including women with previous scars
Group 10	All women with single, cephalic < 37 weeks including women with previous scars

Search strategy and study selection

**Figure 1:**
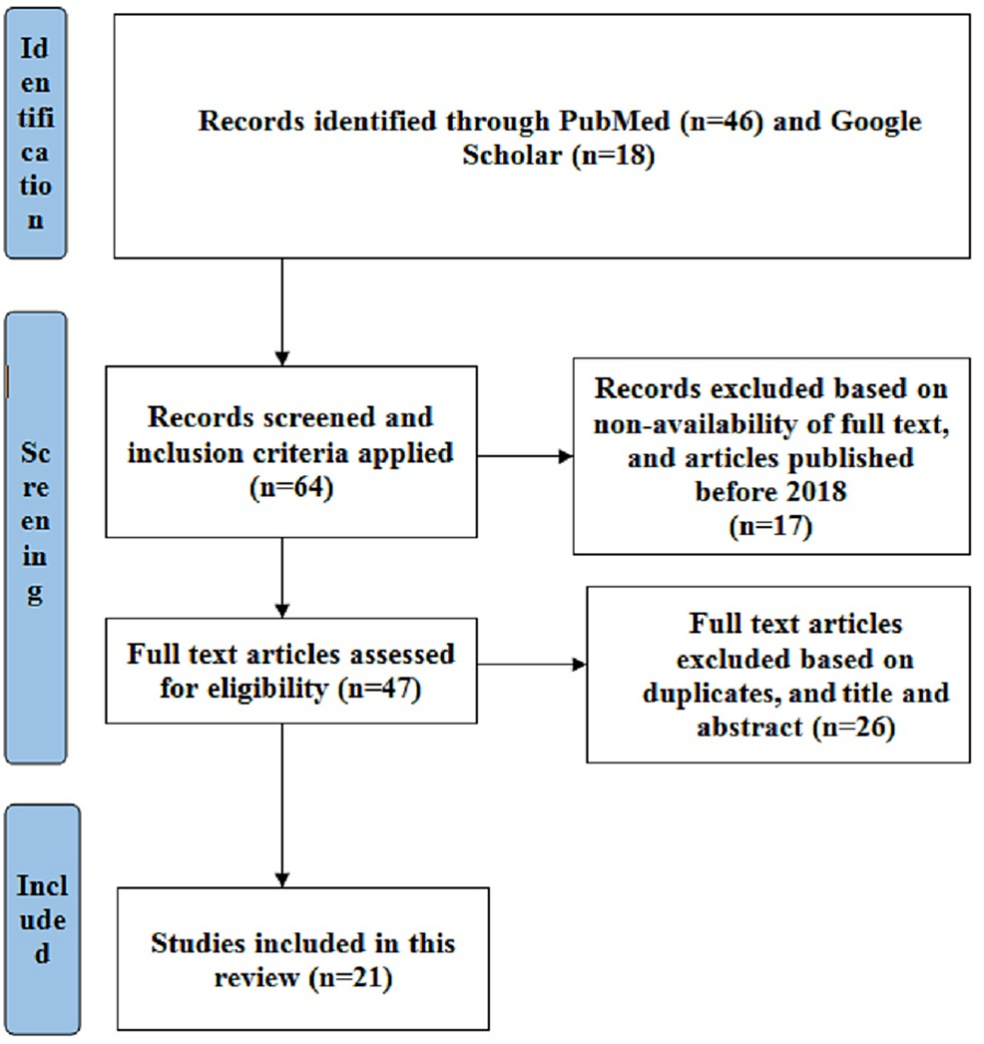
Search strategy for the articles included in the review

Articles that were published from 2018 onwards were included in the search strategy. PubMed and Google Scholar databases were searched using the keywords “Caesarean delivery” OR “Robson’s Ten Group” OR “Classification System”. Sixty-four articles were identified, out of which 17 articles were excluded due to the non-availability of full text and the publication date being prior to 2018. A total of 47 full-text articles met the requirements and were evaluated but only 21 full-text articles were finally included in the current scoping review, which looked at the frequency of caesarean deliveries classified according to Robson's TGCS. This is shown in Figure 1. The details of the 21 articles included in this review are given in Table 2.

**Table 2:** Details of the studies included in the review CS: caesarean section, TGCS: Ten Group Classification System, CD: caesarean delivery, WHO: World Health Organization

Sr. No.	Author and Year	Study design	Methodology	Results
1.	Pravina et al. (2022) [2]	Retrospective study	The study was performed from 2016 to 2019. Women with CS deliveries at this time were tracked down and categorized in accordance with TGCS.	The research's average CS rate was 38.16%, with group 5 contributing the most (34.97%), subsequent to groups 2 (26.35%), 1 (15.51%), and 10 (7.14%).
2.	Parveen et al. (2021) [8]	Cross-sectional study	The study was conducted from 2019 to 2020. Women who underwent CS were recorded and classified into TGCS.	85 individuals, or 50.9% of the total, were found to be from group 10 followed by group 5 consisting of 24 (14.4%), and group 1 consisting of 19 (11.4%) instances. The two most frequent reasons for a CS were found to be fetal discomfort (19.8%) and a previous CS (20.4%).
3.	Tontus et al. (2020) [9]	Retrospective study	The research was carried out over a four-year period. Women having went through CS were noted and placed in the TGCS category.	The total CS prevalence in the research was 51.9%. The CS rate steadily raised from 22.2% in 2013 to 24.3% in 2016.
4.	Gu et al. (2020) [10]	Retrospective study	All CS deliveries were recorded and classified using Robson's TGCS.	Delivery of CS was at a rate of 29.1%. The Robson group 5, which had a CS delivery rate of 80.5%, was the group that participated in the majority of all CS deliveries statistic of 36.6%. CS deliveries were almost twice as common among women in Robson group 2a consisting of Nulliparous, single, cephalic pregnancy > 37 weeks who is labour-induced where the rate was 33.5% compared to group 1's 18.4%.
5.	Crosby et al. (2019) [11]	Retrospective study	Every female was categorized prospectively by applying the obstetric concepts and criteria outlined in the TGCS from 2005 to 2014.	The CS rate increased from 18.3% to 23.5% during the course of ten years and the major contributors were groups 2a consisting of labour-induced, 2b consisting of CS before labour, and 5.
6.	Begum et al. (2019) [1]	Cross-sectional study	In 2015 from June to August, data consisting of case records and operating room registers from 34 urban for-profit private hospitals were involved.	Groups 3 and 4 together made up a size that was 25% larger than the sum of Groups 1 and 2's percentages, which were 15.4%. The percentage of Groups 6 and 7 pregnancies who were breech was 2.7%. At 2.2% and 0.4%, respectively, the number of multiple deliveries in Group 8 and the number of aberrant lays in Group 9 were both substantially under the expected ranges. The group 10 size was higher (30%).
7.	Zimmo et al. (2018) [12]	A population-based birth cohort study.	The overall CS rate, the contribution made by each group, and the rate of each TGCS group were calculated.	Group 5 was the major contributor consisting of 42.6% followed by group 8 (11.6%), and group 10 consisting of 8.1%.
8.	Zeitlin et al. (2021) [13]	Observational study	Numbers of CS in TGCS categories were calculated using data from national statistical agencies and medical birth registrations. In 2015, births that occurred at or before 22 weeks of gestation were all included.	The births through CS ranged from 16.1 to 56.9%. Nations that provided TGCS data had lower rates of CS than those that did not (25.8% vs. 32.9%). However, significant heterogeneity was noted, particularly for groups 5, 6, 7, and 10. The variations in abnormal lie percentages, in group 9, highlight potential misclassification due to inconsistent definitions.
9.	Bracic et al. (2020) [14]	Retrospective Comparative study	The study was performed from 2008-2010. All CS deliveries were recorded and classified using Robson's TGCS.	In these ten years, the CD rate increased from 29.1% to 32.2%. Group 5 was the biggest contributor to the overall CD rate in all examined years, accounting for 20.2% of all CD in 2008–2010 and rising to 26.9% in 2017–2019. Additionally, group 5's overall size raised from 8.3% to 11.6%.
10.	Karalasingam et al. (2020) [15]	Cross-sectional study	The study was conducted from 2011 to 2015. All CS deliveries were recorded and classified using Robson's TGCS.	During the study period, the Caesarean section prevalence was 23.2%. Group 5 had the highest incidence of caesarean sections in general, and the percentage stayed elevated throughout the course of the five-year research.
11.	Matei et al. (2021) [16]	Prospective analysis	This study included every woman less than 20 who gave birth in a tertiary care hospital and was prepared as a one-year prospective evaluation. Prior to discharge, CS delivery patients answered questions about their schooling, prenatal monitoring, and obstetrical history.	47.01% of all births involved CS. 24.57% had at least one previous CS. In middle adolescence, Group 10 was most often identified consisting of 14.03% and in late adolescence, 32.20% were in group 5.
12.	Abubekar et al. (2020) [17]	Cross-sectional study	From January-June 2018 data of all women who delivered through CS were recorded and classified into TGCS.	The total CS incidence was 34.7%. Group 10 (19.1%) was the main predisposing factor, followed by Group 2 (18.3%), Group 5 (17.1%), and Group 4 (15.8%).
13.	Ming et al. (2019) [18]	Cross-sectional study	The research was performed from January to June 2016. A record of total females who delivered through CS was recorded and classified into TGCS.	41.5% of all cases were CS. Group 2b consisting of CS before labor made up the majority of the total CS (37.3%) and among all clinic varieties, was in charge of the second most amount of newborns (15.5%).
14.	Quibel et al. (2020) [19]	Retrospective study	The study was conducted from 2012 to 2018. At a gestational age of ≥24 weeks, all live births were included in the study.	The total CS incidence was 24.5% in the pre-period and 25.1% throughout the post-period.
15.	Liane et al. (2023) [20]	Population-based cohort study.	Data of all women who delivered through CS were recorded and classified into TGCS. CS, intrapartum, perinatal, and neonatal mortality rates expressed as percentages (%) were involved.	In all, the CS rate rose from 12.9% to 16.7% before falling to 15.8% in 2018. The planned CS rate dropped from 9.6% to 4.6% in Robson group 2, whereas the intrapartum CS rate dropped from 26.6% to 22.3%. The planned CS rate dropped from 16.1% to 7.6% and the intrapartum CS rate from 7.8% to 5.2% in Robson group 4.
16.	Sugianto et al. (2022) [21]	Retrospective descriptive study	The study was conducted from January 2018 to December 2018.	The total CS incidence was 34.3%. Among the 73.2% who had no prior experience with CS, group 10 (23.38%) contributed the majority of incidences, next to group 5 (15.84%) and group 2 (13.51%).
17.	Charoonwatana et al. (2022) [22]	Retrospective cross-sectional study	The study was done in 2019. Data of all females who delivered through CS were recorded and classified into TGCS.	The CS rate was 55.4%. The majority 23.3% were in groups 1 to 4, followed by group 5 consisting of 21.1%, and group 10 consisting of 5.4%.
18.	Naik et al. (2021) [23]	Retrospective cross-sectional study	All women who gave birth between December 2019 and November 2020 were enrolled in the present investigation, and their births were analysed and categorised as TGCS.	61.2% consisted of total CS incidences. Group 5, group 2, and group 1 contributed mostly. Groups 6, 7, and 9 did not contribute significantly to the overall CS rate, but the CS rate in this patient population is above 90%, almost approaching 100% in group 6.
19.	Hota et al. (2022) [24]	Retrospective study	The study was conducted for more than five years duration which includes total CS deliveries.	31.39% was the overall CS rate. Group 10 demonstrated a maximum contribution of 31.10% followed by group 5 (29.43%), group 2 (20.95%), and group 1 (06.68%) to the total CS deliveries.
20.	Kore et al. (2021) [25]	Retrospective study	The study was carried out from January 2019 to December 2019. Data of all women who delivered through CS were recorded and classified into TGCS.	In groups 2, 5, and 6 the CS delivery rate was higher than that recommended by WHO.
21.	Konar et al. (2021) [26]	cross-divisional observational study	The study was conducted from 2012 to 2013. Data of all women who delivered through CS were recorded and classified into TGCS.	43.13% was the CS rate. Group 1 is the greater contributor for all the CS incidences which is 15.9% after that group 5 (9.09%), group 3 (5.63%), and group 2 (4.04%).

Discussion

In this review, Liane et al.'s study in 2023 documented a minimal CS rate of 16.2% while Naik et al.'s study in 2021 revealed the greatest CS rate of 61.2% [20,23]. This review's objective was to evaluate the CS delivery percentage according to Robson's TGCS, which is a recognized international system for tracking CS rates. Although the Robson TGCS itself does not reveal the justifications for performing CS, it can serve as a useful beginning point for additional investigation of the Robson group's CS indications if needed [27]. Additionally, this categorization can act as a foundation for a more thorough analysis of all perinatal events and results, as well as the incorporation of epidemiological characteristics [28]. According to Robson et al., TGCS may be used as a typical base of reference to analyse every phase of labour, procedures, and results by including significant epidemiological variables [29].

The review included 21 articles among which 10 studies found that group 5, which included females who had already had a CS and were ≥ 37 weeks pregnant, was the most vulnerable group and the main reason for the overall CS delivery rate. Studies performed in Australia, Canada, and Brazil reported similar outcomes [30-32]. According to the study published by Ming et al., a significant portion of women who had a past CS (96.6%) reported a recurrence of CS [18].

Due to the fact that group 5 performed CS in groups 1-4, which would have resulted in recurrent CS, it is considered to be the main source [9]. Studies presented by Karalasingam et al. and Abubekar et al. reached similar conclusions, concluding that, like many other nations, the rate of CS has increased over time and that this increase is caused by performing CS in low-risk populations [15,17]. The frequency of low-risk nulliparous women who had CS before labour, usually regardless of any known clinical reason, was found to be a major determinant in the high CS rate, according to Ming et al. [18]. These demographics of interest require further in-depth investigation in order to pinpoint possibly adjustable variables and undertake specific measures to reduce the CS rate. To create specialized techniques and enhance outcomes, evaluation of current management protocols and additional research into CS indications and outcomes are required.

According to Bracic et al., the CS rates and population size of this group are on the rise, necessitating the development of more potent strategies to encourage women who have had a previous CS delivery to have a vaginal birth after a CS (VBAC) [14]. The justifications provided for rejecting VBAC included not being able to handle labour pain, unwillingness to agree to a long-term induction in the event of a low Bishop's score, and the conviction that elective repeat CS delivery (ERCD) is a more secure method of the labour, especially for women who have challenging obstetric past events [2]. VBAC is associated with less maternal morbidity and during subsequent pregnancies it reduces the likelihood of complications, consequently decreasing the CS delivery rate. Because they would guarantee that women having VBAC gave their informed permission and participated in decision-making, the Royal College of Obstetricians and Gynaecologists encourages frequent utilisation of VBAC checkpoints during prenatal counselling in order to promote VBAC. Females should be accurately informed of the benefits of VBAC because ERCD is associated with a slightly greater likelihood of placenta previa and/or accreta in upcoming pregnancy, as well as pelvic adhesions compromising any later abdominopelvic surgery [2]. Additionally, the CS delivery rate for preterm births is rising and getting close to 50% [14]. This shows the need for discussion on whether CS is the best delivery method for half of the preterm newborns.

Similarly, in a study given by Crosby et al., the key factor contributing to the increase in CS deliveries was the stimulation of labour and pre-labour CS delivery in nulliparous women having one cephalic conception at term [11]. Similarly to this, the 10-year comparative study by by Bracic et al. found that multiparous women at gestation who had undergone previous CS deliveries were the biggest contributors to CS incidence [14]. According to the study by Parveen et al., group 10 and group 5 births made the largest contributions, while prior CS and fetal distress were the most prevalent symptoms of CS [8]. This obviously leads to a bigger population of women who have already had a CS, which leads to a secondary effect on the rise in the total CS rate [11]. Zimmo et al. found that women in categories 5, 8, and 10 were primarily responsible for the total CS prevalence at the hospitals in which their study took place [12].

Great changes in CS incidence were found among each Robson group, according to the study by Gu et al. [10]. This shows that assessing changes to know the groups (Robson groups 5, 2A, and 1) that contribute to the prevalence of CS deliveries the most might give beneficial knowledge for decreasing CS rates and thus offers a standard for assessing the success of upcoming attempts to cut CS rates in Canada. Contrary to the findings of the previous investigation, Sugianto et al. came to the conclusion that 34.3% of the 385 cases of labour involved CS [21]. Of this, the majority of the CS deliveries were in the women aged 25-29 years, primigravida women, and women without a history of prior CS; the primary gestational age was a term, and the biggest contribution of the CS rate of 23.38% was from Robson group 10 [21]. Furthermore, in a study by Konar et al., the Robson groups 1, 2, 3, and 5 were discovered to be the main drivers of the total CS rate [26]. According to Liane et al., Norway's CS rates rose from 1999 to 2008 before sharply declining from 2008 to 2018 [20]. Fetal and neonatal mortality rates declined at the same time. Obstetricians and midwives from Norway have helped to keep the CS rate around 17%. These results suggest that limiting CS usage is a secure perinatal health strategy.

Additionally, TGCS offers a method for gathering and analyzing data regarding CS rates in an institution. The success of the goal may be determined by doing a thorough review of each group to identify the factors that have contributed to higher CS rates in that group at the institution [23,24]. However, research by Quibel et al. found that the implementation of an audit-and-feedback loop using TGCS did not lower overall CS rates or variation across maternity units [19].

Modifications in education and regulations that establish a common standard among institutions need to be considered since the rise in CS rates reveals a troubling global trend [15]. Increasing the percentage of vaginal deliveries after CS and decreasing primary CS in multiple pregnancies and preterm labour are two ways to diminish the disparities in obstetrical treatment between institutions [12]. To lower the CS rate, these groups could be the focus of successful interventions. Active management of spontaneous onset of labour in a primigravida, lowering the primary CS delivery rate, following CS, and cautious case evaluation prior to inducing labour in nulliparous women are maybe a few helpful therapies [26]. Matei et al. believe that future attention to obstetrical management in Robson groups 7 and 8 is essential [16]. A personal obstetrician and educational level were substantially related to the obstetric recommendation for unnecessary CS, per research by Charoonwatana et al. [22]. The main recipients to lower CS rates are adolescents in Robson group 1 [16].

According to the review, comprehensive research on healthcare institutions classifying CS trend analysis and perinatal auditing using Robson's TGCS is lacking in South Asia (as well as other countries) [9,31-33] and this should be considered as the future scope of the study.

## Conclusions

Group 5 is the major contributing factor to the increase in CS rates, which is followed by groups 10, 4, 2, and 1. Previous CS was the most common factor responsible for increasing CS rates. Hence, Robson’s TGCS is an essential parameter for recording CS and for comparison of CS rates at international, national, state, and institutional levels. For the purpose of optimizing the CS rate, TGCS aids in developing standard policies and strategies aimed at particular categories of women. Encouraging vaginal delivery after CS and lowering first CS should be the major goals of any attempt to decrease the overall CS rate. It is important to regularly analyse signs of CS among key contributors and primary groups and to use consistent and standardised methods.
